# A buffered form of creatine does not promote greater changes in muscle creatine content, body composition, or training adaptations than creatine monohydrate

**DOI:** 10.1186/1550-2783-9-43

**Published:** 2012-09-13

**Authors:** Andrew R Jagim, Jonathan M Oliver, Adam Sanchez, Elfego Galvan, James Fluckey, Steven Riechman, Michael Greenwood, Katherine Kelly, Cynthia Meininger, Christopher Rasmussen, Richard B Kreider

**Affiliations:** 1 Department of Health and Kinesiology, Exercise and Sport Nutrition Laboratory, Texas A&M University, College Station, TX 77843-4243, USA; 2 Department of Sports Medicine and Nutrition, Neuromuscular Research Laboratory, University of Pittsburgh, Pittsburgh, PA, 15203, Oakland; 3 Department of Health and Kinesiology, Muscle Biology Laboratory, Texas A&M University, College Station, TX 77843-4243, USA; 4 Department of Health and Kinesiology, Human Countermeasures Laboratory, Texas A&M University, College Station, TX 77843-4243, USA; 5 Department of Systems Biology and Translational Medicine, Texas A&M Health Science Center, College Station, TX 77843-1114, USA

**Keywords:** Creatine monohydrate, Kre-Alkalyn, Training adaptations, Health, Safety

## Abstract

**Background:**

Creatine monohydrate (CrM) has been consistently reported to increase muscle creatine content and improve high-intensity exercise capacity. However, a number of different forms of creatine have been purported to be more efficacious than CrM. The purpose of this study was to determine if a buffered creatine monohydrate (KA) that has been purported to promote greater creatine retention and training adaptations with fewer side effects at lower doses is more efficacious than CrM supplementation in resistance-trained individuals.

**Methods:**

In a double-blind manner, 36 resistance-trained participants (20.2 ± 2 years, 181 ± 7 cm, 82.1 ± 12 kg, and 14.7 ± 5% body fat) were randomly assigned to supplement their diet with CrM (*Creapure® AlzChem AG, Trostberg, Germany*) at normal loading (4 x 5 g/d for 7-days) and maintenance (5 g/d for 21-days) doses; KA (*Kre-Alkalyn®*, *All American Pharmaceutical, Billings, MT, USA)* at manufacturer’s recommended doses (KA-L, 1.5 g/d for 28-days); or, KA with equivalent loading (4 x 5 g/d for 7-days) and maintenance (5 g/d) doses of CrM (KA-H). Participants were asked to maintain their current training programs and record all workouts. Muscle biopsies from the vastus lateralis, fasting blood samples, body weight, DEXA determined body composition, and Wingate Anaerobic Capacity (WAC) tests were performed at 0, 7, and 28-days while 1RM strength tests were performed at 0 and 28-days. Data were analyzed by a repeated measures multivariate analysis of variance (MANOVA) and are presented as mean ± SD changes from baseline after 7 and 28-days, respectively.

**Results:**

Muscle free creatine content obtained in a subgroup of 25 participants increased in all groups over time (1.4 ± 20.7 and 11.9 ± 24.0 mmol/kg DW, p = 0.03) after 7 and 28-days, respectively, with no significant differences among groups (KA-L −7.9 ± 22.3, 4.7 ± 27.0; KA-H 1.0 ± 12.8, 9.1 ± 23.2; CrM 11.3 ± 23.9, 22.3 ± 21.0 mmol/kg DW, p = 0.46). However, while no overall group differences were observed (p = 0.14), pairwise comparison between the KA-L and CrM groups revealed that changes in muscle creatine content tended to be greater in the CrM group (KA-L −1.1 ± 4.3, CrM 11.2 ± 4.3 mmol/kg DW, p = 0.053 [mean ± SEM]). Although some significant time effects were observed, no significant group x time interactions (p > 0.05) were observed in changes in body mass, fat free mass, fat mass, percent body fat, or total body water; bench press and leg press 1RM strength; WAC mean power, peak power, or total work; serum blood lipids, markers of catabolism and bone status, and serum electrolyte status; or, whole blood makers of lymphocytes and red cells. Serum creatinine levels increased in all groups (p < 0.001) with higher doses of creatine promoting greater increases in serum creatinine (p = 0.03) but the increases observed (0.1 – 0.2 mg/dl) were well within normal values for active individuals (i.e., <1.28 ± 0.2 mg/dl). Serum LDL was decreased to a greater degree following ingesting loading doses in the CrM group but returned to baseline during the maintenance phase. No side effects were reported.

**Conclusions:**

Neither manufacturers recommended doses of KA (1.5 g/d) or KA with equivalent loading (20 g/d for 7-days) and maintenance doses (5 g/d for 21-days) of CrM promoted greater changes in muscle creatine content, body composition, strength, or anaerobic capacity than CrM (20 g/d for 7-days, 5 g/d for 21-days). There was no evidence that supplementing the diet with a buffered form of creatine resulted in fewer side effects than CrM. These findings do not support claims that consuming a buffered form of creatine is a more efficacious and/or safer form of creatine to consume than creatine monohydrate.

## Background

Creatine has proven to be one of the most effective and popular dietary supplements for resistance-trained athletes [1-3]. The form of creatine that has been most extensively studied has been creatine monohydrate (CrM) [1]. Studies have consistently indicated that creatine supplementation increases muscle creatine and phosphocreatine concentrations by approximately 15-40%, enhances anaerobic exercise capacity, and increases training volume leading to greater gains in strength, power, and muscle mass [1-10]. A number of potential therapeutic benefits have also been suggested in various clinical populations [11-17]. Studies have indicated that creatine monohydrate is not degraded during normal digestion and that nearly 99% of orally ingested creatine is either taken up by tissues or excreted in urine [18-20]. Further, no medically significant side effects have been reported in the literature [21-27]. Nevertheless, supplement manufacturers have continually introduced newer forms of creatine into the marketplace [1]. These newer forms have been purported to have better physical and chemical properties, bioavailability, efficacy, and/or safety profiles than creatine monohydrate [1]. However, there is little to no evidence that any of the newer forms of creatine are more effective and/or a safer form of creatine than CrM whether ingested alone and/or in combination with other nutrients [1]. In addition, whereas the safety, efficacy, and regulatory status of CrM is clearly defined in almost all global markets; the safety, efficacy and regulatory status of other forms of creatine present in today’s marketplace as a dietary or food supplement is less clear [1].

A buffered form of creatine *(Kre-Alkalyn® [KA], All American Pharmaceutical, Billings, MT, USA)* has been marketed as a more efficacious and safer form of creatine than creatine monohydrate [28]. According to the manufacturer’s website [28], this patented form of creatine [29] is a “*buffered*” or “*pH-correct*” form of creatine that remains more stable in the stomach, is not degraded to creatinine, and thereby has greater bioavailability. According to patent filings [29], this is accomplished by adding an alkaline powder (e.g., soda ash, magnesium glycerol phosphate, bicarbonate) to creatine (e.g., creatine monohydrate, creatine phosphate, creatine pyruvate, creatine citrate) in order to adjust the pH to a range between 7–14. The manufacturer claims that this form of creatine is “*the only Creatine guaranteed to stay 100% stable all the way to the muscle cell*”; that it is “*up to ten times more powerful than ordinary Creatine*”; that “*1.5 grams of Kre-Alkalyn is equivalent to about 10–15 grams of ordinary Creatine*”; that it is “*an alternative to all the bloating, cramping, and other side effects associated with traditional creatine supplementation*”; and, that it is “*the world’s most potent creatine*” [28]. The manufacturer cites several clinical studies on their website performed in Bulgaria to support their claims [28,30]. However, we could find no peer-reviewed articles cited in the National Library of Medicine’s PubMed related to “*Kre-Alkalyn”,* or *“buffered creatine”* from the purported study authors or anyone else. One paper that was presented at the International Society of Sports Nutrition annual meeting in 2007 reported that the conversion of creatine to creatinine from CrM at a pH of 1.0 and 37°C was less than 1% after 5, 30 and 120 minutes while KA had a 35% greater conversion to creatinine under similar conditions [31]. However, full details of this study have yet to be published.

Our research group has extensive experience in conducting clinical research studies on the efficacy and safety of supplementing the diet during training with various forms of creatine [9,25,26,32-39]. As a result, AlzChem AG (*Trostberg, Germany*), a primary raw material provider of pure creatine monohydrate, provided a grant to our university to conduct an independent research study to compare the effects of supplementing the diet with KA at recommended doses (1.5 g/d for 28-days) and creatine equivalent loading (20 g/d for 7-days) and maintenance doses (5 g/d for 21-days) of KA to CrM (20 g/d for 7-days, 5 g/d for 21-days) on muscle creatine retention, body composition, strength, anaerobic capacity and markers of health status. We also sought to determine whether ingesting the purported buffered form of creatine would be associated with fewer side effects than creatine monohydrate as claimed. Theoretically, if KA is indeed a more efficacious form of creatine, the recommended doses of KA (1.5 g/d) would be as effective or more effective than consuming standard loading (20 g/d for 7-day) and maintenance doses (5 g/d for 21-days) of CrM on increasing muscle creatine levels and training adaptations with fewer side effects. Additionally, ingesting creatine equivalent loading and maintenance doses of KA would theoretically promote greater effects with fewer side effects in those ingesting standard loading and maintenance doses of CrM.

## Methods

### Experimental design

Table 1 presents the general experimental design employed in this study. The study was conducted in a double-blind, randomized controlled manner. The independent variable was the type of creatine ingested. Dependent variables included muscle creatine content, body composition, one repetition maximum (1RM) bench press and leg press, anaerobic sprint performance capacity, serum and whole blood clinical markers of health, and self-reported side effects. Dietary intake was not controlled but participant’s dietary intake was recorded prior to each testing session and analyzed for energy intake and macronutrient content. Participants were instructed to maintain their normal resistance-training program and maintain training logs so training volume could be compared. Subjects who qualified for the study participated in a familiarization session in which the study was explained to the participants and informed consent was obtained. After the familiarization session, subjects were matched for bodyweight, years of training experience, and age and randomly assigned to one of three groups: 1.) KA at manufacturer’s recommended doses (KA-L, 1.5 g/d for 28-days); 2.) KA at creatine equivalent loading (4 x 5 g/d for 7-days) and maintenance (5 g/d for 21-days) doses as CrM (KA-H); or, 3.) CrM at normal loading (4 x 5 g/d for 7-days) and maintenance doses (5 g/d for 21-days).

**Table 1:** Overview of Study Design

**Familiarization and Entry**	**Baseline Day 0**	**Loading Phase Day 7**	**Maintenance Phase Day 28**
Familiarization session	4-Day Diet History	4-Day Diet History	4-Day Diet History
Informed Consent Form	Muscle Biopsy	Submit Training Log	Submit Training Log
Demographic Form	Fasting Blood Sample Body Weight	Muscle Biopsy	Muscle Biopsy
Health History Form	Body Water (BIA)	Fasting Blood Sample	Fasting Blood Sample
Exercise History Form	DEXA Body Composition	Body Weight	Body Weight
4-day Dietary History	1 RM Leg Press	Body Water (BIA)	Body Water (BIA)
General Exam to Determine Qualifications to Participate in Study	1 RM Bench Press	DEXA Body Composition	DEXA Body Composition
Height and Body Weight	Wingate Anaerobic Capacity Test	Wingate Anaerobic Capacity Test	1 RM Leg Press
Practice Wingate Anaerobic Capacity Test	Loading Phase of Supplementation Begins	Low-Dose Maintenance Phase of Supplementation Begins	1 RM Bench Press
Randomization into one of three groups (CrM, KA-L, KA-H)	Maintain Training Log		Wingate Anaerobic Capacity Test
Instructions for Supplementation			

### Participants

Apparently healthy resistance-trained males with no self-reported recent history of creatine supplementation were recruited to participate in this study. Participants were not allowed to participate in this study if they had any metabolic disorder including known electrolyte abnormalities; heart disease, arrhythmias, diabetes, thyroid disease, or hypogonadism; a history of hypertension, hepatorenal, musculoskeletal, autoimmune, or neurologic disease; if they were taking thyroid, anti-hyperlipidemic, hypoglycemic, anti-hypertensive, anti-inflammatory, or androgenic medications; or, if they had taken dietary supplements containing creatine within three months prior to the start of the study. Participants were recruited from the student population and from area fitness facilities. Participants completed demographic, health history and exercise history forms. Those who met eligibility criteria were informed of the requirements of the study and signed informed consent statements in compliance with the Human Subjects Guidelines of Texas A&M University and the American College of Sports Medicine. Subjects participated in a familiarization session that included practicing the Wingate anaerobic capacity test.

### Testing sessions

Participants were instructed to record all food ingestion on food record forms four days (4-d) prior to the start of the study. In addition, subjects were asked to fast for 8 hours and abstain from exercise for 48 hours prior to baseline testing. Once reporting to the lab, subjects donated a muscle biopsy and fasting blood samples using standard clinical procedures. Subjects were then weighed, had body water assessed using a bioelectrical impedance analyzer (BIA), and body composition assessed using a Dual-Energy X-Ray Absorptiometer (DEXA). They also performed 1RM tests on the bench press and hip sled/leg press and performed a 30-second Wingate anaerobic capacity sprint test on a cycle ergometer. Subjects then began a 7-day initial supplementation phase. After 7 days, subjects repeated all tests with the exception of 1RM strength measures. The subjects then followed supplementation schedules for 21-days and returned to undergo all tests. This allowed for the assessment of acute and chronic supplementation protocols on muscle creatine levels, body composition, exercise performance, as well as markers of clinical health and safety. Participants were asked to maintain their current training programs and record all workouts. Participants were also asked to report side effects on a weekly basis.

### Supplementation protocol

Participants were matched into one of three groups according to body weight, training status/experience, and age. Subjects were then randomly assigned to one of three groups to ingest, in a double blind manner, capsules containing CrM (*Creapure® AlzChem AG, Trostberg, Germany, Lot #108631*) or KA *(Kre-Alkalyn® All American Pharmaceutical, Billings, MT, USA, Lot #1067000)* at two different dosages. Supplements were provided by the supporting sponsor in red 0.75 gram (00 sized) capsules and placed in generic single-serving packets that were put in labeled containers for double-blind administration on a weekly basis. Creatine content of the capsules was independently verified by Covance Laboratories (*Madison, WI*). Certificate of analysis results are presented in Table 2. Participants in the CrM groups ingested 8 capsules per serving containing approximately 5 g of CrM four times daily (20 g/d) for 7-days and once per day (5 g/d) for 21-days. A small amount of dextrose (~60 mg per capsule) was added to the CrM capsules to enhance flowability during encapsulation. Participants in the KA creatine monohydrate equivalent group (KA-H) ingested 8 capsules per serving containing approximately 5 g of CrM four times daily (20 g/d) for 7-days and once per day (5 g/d) for 21-days. Participants assigned to ingest the manufacturers recommended doses of KA (KA-L) ingested 8 capsules containing a total of approximately 1.5 g of KA mixed with 3.5 g of dextrose once per day and 8 capsules containing 5 g of dextrose three times per day during the initial 7-day loading period. Thereafter, participants in the KA-L group ingested 8 capsules per day containing 1.5 g/d of KA mixed with 3.5 g of dextrose for 21-days. Participants were instructed to ingest supplements at 8:00 am, 12:00 pm, 4:00 pm, and 8:00 pm during the initial 7-day supplementation period and at 8:00 am during the maintenance phase. Supplementation compliance was monitored by having the subjects return empty containers of the supplements at the end of each week. In addition, subject’s compliance was verified by administering and collecting weekly questionnaires. After completing the compliance procedures, the subjects were given the required supplements for the next week.

**Table 2:** Supplement Certificate of Analysis Results

**Group**	**Entity Weight (g)**	**Fill Weight (g)**	**Moisture (%)**	**Creatine Monohydrate (%)**	**Total Creatine Monohydrate (g/per 8 capsules)**	**Creatinine (ppm)**
KA-L	0.7609	0.6375	8.2	30.6	1.56	<5,000
KA-H	0.7566	0.6358	8.8	102.0	5.19	<5,000
CrM	0.8171	0.6975	9.4	92.4	5.16	<5,000

Samples analyzed by Covance Laboratory Inc. (*Madison, WI*). Sample size was eight capsules.

### Procedures

#### Diet and training analysis

Participants were instructed to maintain their current dietary habits and to keep detailed dietary records. Prior to each testing session subjects completed a dietary record that included 3 weekdays and 1 weekend day. Dietary inventories were reviewed by a registered dietitian and analyzed for average energy and macronutrient intake using the Food Processor Nutrition Analysis Software Version 9.1.0 (*ESHA Nutrition Research, Salem, OR*). Participants were also instructed to maintain their current training regimen and record the type and number of sets and repetitions performed on training logs. Training volume was calculated by multiplying the amount of weight lifted times the number of repetitions performed for each set performed. Total training volume during the study was analyzed by summing all lifts (upper and lower body) to determine if there were any differences among groups.

#### Body composition

Body composition testing occurred on day 0, 7 and 28 of the study. Height and weight were recorded to the nearest 0.02 kg and 0.01 cm, respectively, using a self-calibrating digital scale (*Cardinal Detecto Scale Model 8430, Webb City, Missouri*). Body composition was determined using a Hologic Discovery W QDR series DEXA system (*Hologic Inc., Waltham, MA*) equipped with APEX software (*APEX Corporation Software version 12.1, Pittsburgh, PA*). Quality control calibration procedures were performed on a spine phantom (Hologic-X-CLAIBER Model DPA/QDR-1 anthropometric spine phantom) and a density step calibration phantom prior to each testing session. DEXA has been validated as an accurate method for body composition assessment [40]. Mean test-retest reliability studies performed on male athletes in our lab has yielded mean coefficients of variation for total bone mineral content and total fat free/soft tissue mass of 0.31% to 0.45% with a mean intra-class correlation of 0.985 [41]. Body water was estimated using an ImpediMed DF50 bioelectrical impedance analyzer (*ImpediMed, San Diego, CA*).

#### Blood and muscle samples

Subjects donated approximately 10 ml of fasting blood using venipuncture techniques from an antecubital vein in the forearm according to standard sterile procedures. Serum blood samples were sent to Quest Diagnostics (*Houston, TX*) for comprehensive metabolic panel analysis using an Olympus AAU 5400 Chemistry Immuno Analyzer (*Olympus America Inc.,Center Valley, PA*). Whole blood samples were analyzed for complete blood counts with platelet differentials using an Abbott Cell Dyn 3500 automated hematology analyzer (*Abbott Laboratories, Abbott Park, IL*). Reported test to test reliability of performing these assays generally range from 2 to 6% for individual assays. Samples were run in duplicate to verify results if the observed values were outside control values and/or clinical norms according to standard procedures.

Muscle biopsies were obtained using a modified Bergstrom needle biopsy technique following standard procedures [42]. Percutaneous muscle biopsies (50–70 mg) were obtained from the middle portion of the vastus lateralis muscle of the dominant leg at the midpoint between the patella and the greater trochanter of the femur at a depth between 1 and 2 cm into the muscle. For the remaining two biopsies, attempts were made to extract tissue from approximately the same location as the initial biopsy by using the pre-biopsy scar, depth markings on the needle, and successive incisions that were made approximately 2 cm proximal to the former site. After removal, adipose tissue was trimmed from the muscle specimens which were then immediately frozen in liquid nitrogen and then stored at −80°C for later analysis. A total of three muscle samples were obtained (Day 0, 7, & 28). Muscle tissue samples were analyzed spectrophotometrically in duplicate for creatine (Cr) using methods developed by Harris and colleagues [7,8,43]. Briefly, approximately 50–70 mg of muscle tissue was cut and placed in a microfuge tube, and then placed in a vacuum centrifuge (*Savant ISS110 SpeedVac Concentrator, Thermo Scientific, Milford, MA*) and centrifuged for 18–24 hours. Connective tissue was removed from the dried samples which were then grinded into a powder in a porcelain plate and placed into pre-weighed microfuge tubes. Muscle metabolites were extracted in a 0.5 M perchloric acid/ 1 mM EDTA solution on ice for 15 minutes, while periodically vortexing. Samples were then centrifuged at 7,000 rpm for 5 minutes. The supernatant was transferred into a pre-weighed microfuge tube and neutralized with 2.1 M KHCO3/0.3 M MOPS solution. The samples were then centrifuged again at 7,000 rpm for 5 minutes and the supernatant was removed and placed into microfuge tubes and frozen at −80°C.

Extracts were assayed for Cr in the presence of 50 mM imidazole buffer, pH 4.7; 5 mM magnesium chloride; 20 mM potassium chloride; 25 μM phosphoenolpyruvate; 200 μM ATP; 45 μM NADH; 1250 U/mL lactate dehydrogenase; 2000 U/mL pyruvate kinase. The assay was carried out in a standard fluorescence microplate reader using 10 μL of sample to 1 mL of reagent. The reactant solution was vortexed and read using a fluorometer (*Shimadzu RFMini 150, Japan*) with an excitation wavelength of 340 nm and an emission wavelength of 460 nm for baseline absorbance values. Five μL of CK (25 μ/mg) was added to 1 mL of the above buffer and stabilized using 1 mL of reagent. After 10 minutes the plate was read again for post-reaction absorbance values. Test to test reliability of duplicate muscle creatine assays was 0.22 ± 2.4% (r = 0.90) with a coefficient of variation of 6.8. We also assayed muscle samples for phosphocreatine (PCr) but several values were out of normal ranges, there was large variability in values observed, and overall PCr levels declined over time despite creatine supplementation suggesting a lack of validity in this assay. Therefore, these data were not reported.

#### Performance tests

Maximal strength tests were performed using a standard isotonic Olympic bench press and hip sled/leg press (*Nebula Fitness, Versailles, OH*) according to standardized procedures [44]. Hand positioning on the bench press and foot and seat position on the hip sled/leg press were standardized between trials. Participants followed a standardized warm-up (10 repetitions at 50% of 1RM) prior to beginning 1RM attempts. Rest recovery was standardized between attempts at 2-min and participants typically reached their 1RM within 3–5 attempts after warming up. Participants performed the hip sled/leg press 1RM test, rested for 4 minutes, and then began warming up on the bench press. Bench press 1RM was determined following similar procedures as the hip sled/leg press 1RM test. Test-to-test reliability of performing these tests in our lab on resistance-trained participants have yielded low day to day mean coefficients of variation and high reliability for the bench press (1.1%, intra-class r = 0.99) and hip sled/leg press (0.7%, intra-class r = 0.91). Subjects rested for about 20-minutes and then warmed up on a bicycle ergometer for 3-minutes (70 rpm @ 1 kg resistance). Participants then performed a 30-second Wingate sprint anaerobic capacity test on a Lode Excalibur Sport 925900 cycle ergometer (*Lode BV, Groningen, The Netherlands*) at a standardized work rate of 7.5 J/kg/rev. The seat position was standardized between trials and the participant was asked to pedal as fast as possible prior to application of the workload and sprint at all-out maximal capacity during the 30-second test. Test-to-test variability in performing repeated Wingate anaerobic capacity tests in our laboratory yielded correlation coefficients of *r =* 0.98 ±15% for mean power. Participants practiced the anaerobic capacity test during the familiarization session to minimize learning effects.

#### Side effect assessment

Participants were given weekly questionnaires on how well they tolerated the supplement, how well they followed the supplement protocol, and if they experienced any medical problems/symptoms during the study. Compliance to the supplementation protocol was monitored by turning in empty weekly supplement containers, supplement logs and verbal confirmation. After completing the compliance procedures, subjects were given the required supplements and dosages for the following supplementation period.

#### Data analysis

Participant baseline demographic data were analyzed by one-way Analysis of Variance (ANOVA). Study data were analyzed by Multivariate Analysis of Variance (MANOVA) with repeated measures. Overall MANOVA effects were examined using the Wilks’ Lamda time and group x time p-levels as well as MANOVA univariate ANOVA group effects. Greenhouse-Geisser univariate tests of within-subjects time and group x time effects and between-subjects univariate group effects were reported for each variable analyzed within the MANOVA model. In some instances, repeated measures ANOVA was run on variables not included in a MANOVA design with univariate group, time, and group x time interaction effects reported. Data were considered statistically significant when the probability of type I error was 0.05 or less and statistical trends were considered when the probability of error ranged between p > 0.05 to p < 0.10. If a significant group, treatment and/or interaction alpha level was observed, Tukey’s least significant differences (LSD) post-hoc analysis was performed to determine where significance was obtained. *A priori* power analysis of the design indicated that an n-size of 12 per group was sufficiently powered to identify previously reported changes in muscle creatine content and training adaptations in responses to creatine supplementation (>0.70).

## Results

### Subject demographics

Forty-one participants were initially recruited for the study, completed consent forms and participated in the required familiarization session. Of the original 41 participants, 36 completed the 28-day research study. Three participants dropped out due to time constraints, one due to an unrelated illness, and one due to apprehension of the muscle biopsy procedure. None of the participants dropped out of the study due to side effects related to the study protocol. Table 3 shows the baseline demographics for the participants. Overall, participants were 20.2 ± 2 years, 181 ± 7 cm, 82.1 ± 12 kg, and 14.7 ± 5% fat with 3.8 ± 3 years of resistance training experience. One-way ANOVA revealed no significant differences among groups in baseline demographic variables.

**Table 3:** Participant Demographics

**Group**	**N**	**Age (years)**	**Height (cm)**	**Body Weight (kg)**	**Body Fat (%)**	**Training (years)**
KA-L	12	19.8±1.8	180.1±8.4	83.4±13.6	17.0±4.9	3.0±2.5
KA-H	12	19.5±1.2	181.0±6.3	81.2±8.1	12.8±4.1	4.0±2.9
CrM	12	21.3±2.8	181.3±6.4	81.8±13.8	14.2±4.7	4.3±3.4
p-level		0.07	0.91	0.90	0.08	0.55

Values are means ± standard deviations.

Data were analyzed by one-way ANOVA.

### Compliance, side effects, training, and diet

Based on compliance records, all participants exhibited 100% compliance with the supplementation protocol without experiencing any side effects throughout the duration of the 28-day supplementation protocol. Table 4 shows the total training volumes for upper and lower body lifts. One-way ANOVA revealed that there were no significant differences among groups in total upper body training volume (p = 0.89) or lower body training volume (p = 0.55). Table 5 presents mean energy intake and macronutrient content for each group. MANOVA revealed no overall significant Wilks’ Lambda time (p = 0.39) or group x time (p = 0.56) interaction effects in absolute energy intake (kcal/d), protein intake (g/d), carbohydrate (g/d) or fat intake (g/d). MANOVA univariate analysis revealed a significant time effect suggesting that energy and protein intake tended to decrease during the study but no significant interactions were observed among groups. Similar results were observed when assessing energy and macronutrient intake when expressed relative to body mass.

**Table 4:** Training Volume

**Group**	**Upper Body (kg)**	**Lower Body (kg)**
KA-L	65,006 ± 35,543	40,631 ± 20,641
KA-H	74,445 ± 42,340	32,930 ± 20,258
CrM	69,227 ± 62,251	32,665 ± 19,471
p-level	0.89	0.55

Training logs were obtained on all participants (n = 36 or 12 per group).

Values are means ± standard deviations.

Data were analyzed by one-way ANOVA.

**Table 5:** Dietary Caloric and Macronutrient Intake

**Variable**	**Group**	**Day**				**p-level**
		**0**	**7**	**28**		
**Calories** (kcal/day)	KA-L	2,167 ± 900	2,202 ± 653	1,998 ± 444	Group	0.29
	KA-H	2,506 ± 645	2,604 ± 670	2,321 ± 677	Time	0.08
	CrM	2,511 ± 582	2,372 ± 735	2,312 ± 394	G x T	0.81
**Protein** (g/d)	KA-L	126.3 ± 76	126.2 ± 58	112.4 ± 46	Group	0.65
	KA-H	139.4 ± 46	143.2 ± 54	132.5 ± 60	Time	0.05
	CrM	127.8 ± 28	131.2 ± 40	114.1 ± 35	G x T	0.97
**Carbohydrate** (g/d)	KA-L	219.1 ± 73	203.9 ± 79	181.7 ± 53	Group	0.53
	KA-H	221.9 ± 74	216.0 ± 91	206.1 ± 86	Time	0.40
	CrM	231.0 ± 72	226.1 ± 93	242.6 ± 66	G x T	0.38
**Fat** (g/d)	KA-L	78.6 ± 38	84.7 ± 27	71.6 ± 16	Group	0.20
	KA-H	99.2 ± 40	105.7 ± 47	94.5 ± 35	Time	0.19
	CrM	91.3 ± 32	81.3 ± 30	83.0 ± 20	G x T	0.47
**Calories**	KA-L	26.2 ± 10.0	26.6 ± 7.9	24.4 ± 7.2	Group	0.29
(kcal/kg/d)	KA-H	31.4 ± 9.5	32.1 ± 10.5	28.3 ± 9.4	Time	0.06
	CrM	31.2 ± 7.5	29.0 ± 8.8	28.4 ± 5.8	G x T	0.73
**Protein**	KA-L	1.50 ± 0.8	1.52 ± 0.7	1.36 ± 0.6	Group	0.58
(g/kg/d)	KA-H	1.75 ± 0.7	1.76 ± 0.8	1.61 ± 0.8	Time	0.04
	CrM	1.59 ± 0.4	1.61 ± 46	1.41 ± 0.4	G x T	0.99
**Carbohydrate**	KA-L	2.69 ± 1.0	2.48 ± 0.9	2.21 ± 0.7	Group	0.50
(g/kg/d)	KA-H	2.75 ± 0.9	2.65 ± 1.2	2.46 ± 1.0	Time	0.24
	CrM	2.87 ± 0.9	2.76 ± 1.1	2.99 ± 0.9	G x T	0.34
**Fat**	KA-L	0.96 ± 0.4	1.02 ± 0.3	0.87 ± 0.2	Group	0.23
(g/kg/d)	KA-H	1.24 ± 0.6	1.31 ± 0.7	1.16 ± 0.5	Time	0.14
	CrM	1.14 ± 0.4	1.0 ± 0.4	1.01 ± 0.3	G x T	0.44

Nutritional records were analyzed on all participants (n = 36 or 12 per group). Values are means ± standard deviations. Absolute and relative nutritional data were analyzed by MANOVA. Greenhouse-Geisser time and group x time (G x T) interaction p-levels are reported with univariate group p-levels.

### Muscle creatine analysis

**Figure 1:**
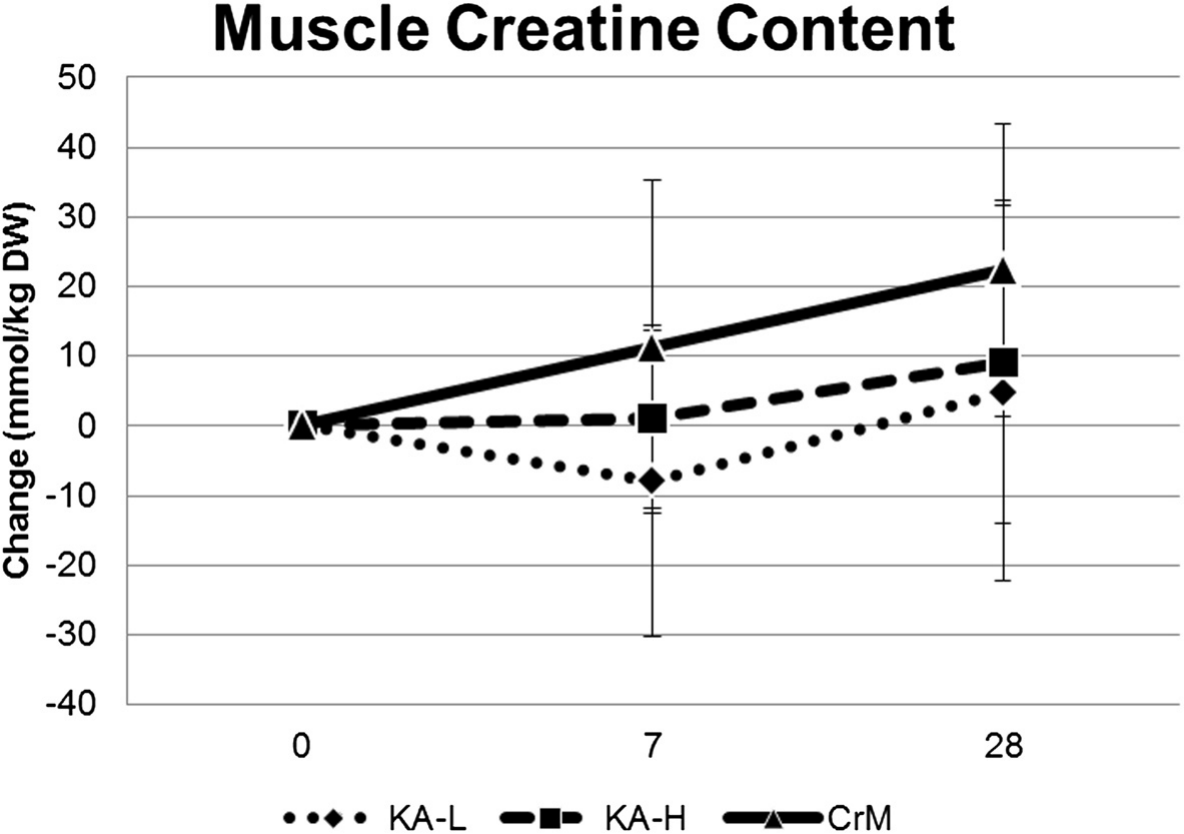
Changes in muscle free creatine content from baseline.

Table 6 presents muscle free creatine content data while Figure 1 shows changes in muscle free content. Sufficient muscle samples were obtained to measure baseline and subsequent creatine on 25 participants. Subjects with missing baseline or day-28 data were not included in the analysis. Two day-7 missing creatine values were replaced using the last observed value method. A MANOVA was run on muscle creatine expressed in mmol/kg DW and changes from baseline expressed in mmol/kg DW and percent changes from baseline. An overall MANOVA time effect (Wilks’ Lamda p = 0.002) was observed with no significant overall MANOVA group x time interactions (Wilks’ Lambda p = 0.55). MANOVA univariate analysis revealed significant time effects in muscle free creatine content expressed in absolute terms (p = 0.03), changes from baseline (p = 0.03), and percent changes from baseline (p = 0.003). No significant groups x time interactions were observed among groups. However, while no overall group differences were observed (p = 0.14), pairwise comparison between the KA-L and CrM groups revealed that changes in muscle creatine tended to be greater in the CrM group (KA-L −1.1 ± 4.3, CrM 11.2 ± 4.3 mmol/kg DW, p = 0.053 [mean ± SEM]; KA-L 2.4 ± 8.5, CrM 24.6 ± 8.5%, p = 0.078 [mean ± SEM]).

**Table 6:** Muscle Creatine Levels

**Variable**	**N**	**Group**	**Day**				**p-level**
			**0**	**7**	**28**		
**Cr** (mmol/kg DW)	8	KA-L	65.8 ± 15.4	57.9 ± 16.1	70.5 ± 20.9	Group	0.74
	9	KA-H	57.3 ± 17.7	58.3 ± 15.6	66.3 ± 12.6	Time	0.03
	8	CrM	51.5 ± 12.7	62.8 ± 25.0	73.8 ± 15.6	G x T	0.46
**Cr**	8	KA-L	0.0 ± 0.0	−8.0 ± 22.3	4.71 ± 27.0	Group	0.14
(Δ mmol/kg DW)	9	KA-H	0.0 ± 0.0	1.03 ± 12.8	9.07 ± 23.2	Time	0.03
	8	CrM	0.0 ± 0.0	11.3 ± 23.9	22.3 ± 21.0	G x T	0.46
**Cr** (Δ %)	8	KA-L	0.0 ± 0.0	−6.4 ± 37.8	13.7 ± 42.2	Group	0.20
	9	KA-H	0.0 ± 0.0	6.2 ± 29.2	27.3 ± 49.1	Time	0.003
	8	CrM	0.0 ± 0.0	23.5 ± 49.0	50.4 ± 44.8	G x T	0.51

Values are means ± standard deviations. Δ represents change from baseline values. Sufficient muscle samples were obtained to measure baseline and subsequent Cr on 25 participants. Missing day-7 data in participants with baseline and day-28 values were replaced using the last observed value method (n = 2). Data were analyzed by MANOVA with repeated measures. Greenhouse-Geisser time and group x time (G x T) interaction p-levels are reported with univariate group p-levels.

### Body composition

**Figure 2:**
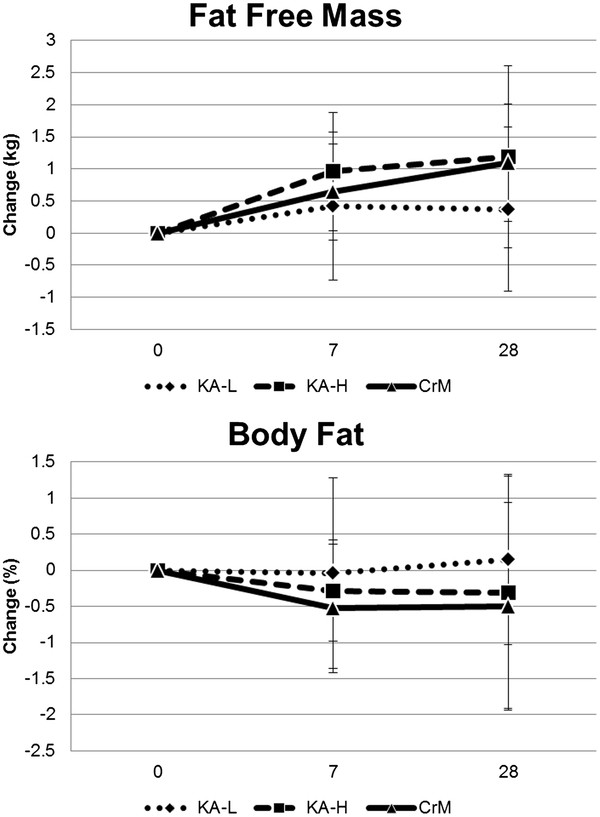
Changes in fat free mass and body fat from baseline.

Table 7 presents body composition results observed during the study while Figure 2 shows the changes observed over time in fat free mass and percent body fat. Overall MANOVA revealed significant time effects (Wilks’ Lambda p = 0.001) with no significant group x time interactions observed (Wilks’ Lambda p = 0.90) in body composition variables. Bodyweight increased in all groups over time (1.0 ± 1.9, 1.42 ± 2.5 kg, p < 0.001) with no significant group x time interaction effects observed among groups after 7 and 28-days, respectively, of supplementation (KA-L 0.7 ± 0.83, 0.9 ± 1.6; KA-H 1.7 ± 2.9, 2.3 ± 3.7; CrM 0.6 ± 1.1, 1.1 ± 1.4 kg, p = 0.35). Fat-free mass significantly increased over time for all groups (0.67 ± 1.0, 0.89 ± 1.2 kg, p < 0.001) with no significant group x time interaction effects observed among groups (KA-L 0.42 ± 1.2, 0.37 ± 1.3; KA-H 0.96 ± 0.9, 1.2 ± 1.4; CrM 0.6 ± 0.8, 1.1 ± 0.9 kg, p = 0.43). Body fat percent was not significantly decreased over time for all groups (−0.28 ± 1.0, -0.22 ± 1.4%, p = 0.41) and no significant group x time interactions were observed among groups (KA-L −0.04 ± 1.3, 0.15 ± 1.2; KA-H −0.28 ± 0.7, -0.31 ± 1.6; CrM −0.53 ± 0.9, -0.50 ± 1.4%, p = 0.77). Total body water expressed as a percentage of bodyweight significantly decreased over time for all groups (−1.25 ± 3.7, -2.68 ± 3.4%, p < 0.001) with no significant group x time interaction effects observed among groups (KA-L −0.58 ± 4.1, -1.95 ± 4.4; KA-H −2.25 ± 2.0, -3.28 ± 3.1; CrM −0.92 ± 4.6, -2.82 ± 2.6%, p = 0.71).

**Table 7:** Body Composition

**Marker**	**Group**	**Day**				**p-level**
		**0**	**7**	**28**		
**Body Weight** (kg)	KA-L	83.4 ± 13.6	84.1 ± 14.0	84.3 ± 13.6	Group	0.94
	KA-H	81.2 ± 8.1	83.0 ± 9.7	83.5 ± 10.3	Time	0.001
	CrM	81.8 ± 13.8	82.3 ± 13.6	82.9 ± 13.0	G x T	0.35
**Fat Mass** (kg)	KA-L	13.5 ± 5.4	13.7 ± 5.9	13.8 ± 5.8	Group	0.11
	KA-H	9.7 ± 3.2	9.6 ± 3.1	9.6 ± 3.1	Time	0.82
	CrM	11.0 ± 5.3	10.7 ± 5.4	10.6 ± 4.4	G x T	0.73
**Fat-Free Mass** (kg)	KA-L	61.3 ± 8.7	61.7 ± 8.6	61.7 ± 8.8	Group	0.77
	KA-H	63.5 ± 8.0	64.4 ± 8.0	64.7 ± 8.4	Time	0.001
	CrM	62.3 ± 9.8	63.0 ± 9.6	63.4 ± 9.9	G x T	0.43
**Body Fat Percent** (%)	KA-L	17.0 ± 4.9	17.0 ± 5.5	17.2 ± 5.4	Group	0.06
	KA-H	12.8 ± 4.1	12.5 ± 3.8	12.5 ± 3.6	Time	0.41
	CrM	14.2 ± 4.7	13.7 ± 5.0	13.7 ± 4.2	G x T	0.77
**Total Body Water** (%)	KA-L	37.8 ± 5.0	37.2 ± 4.4	35.9 ± 3.3	Group	0.26
	KA-H	37.4 ± 2.9	35.1 ± 2.6	34.1 ± 1.7	Time	0.00
	CrM	36.7 ± 2.7	35.8 ± 3.0	33.9 ± 1.5	G x T	0.71

Values are means ± standard deviations. DEXA body composition and BIA determined body water were determined on 36 participants (12 per group). Body composition variables were analyzed by MANOVA with repeated measures. Greenhouse-Geisser time and group x time (G x T) interaction p-levels are reported with univariate group p-levels.

### Training adaptations

**Figure 3:**
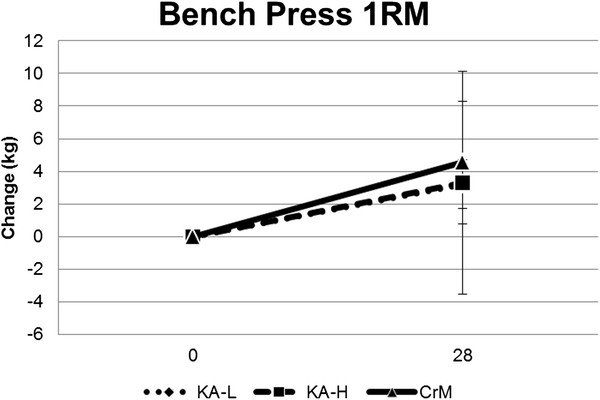
Changes in bench press 1RM strength from baseline.

**Figure 4:**
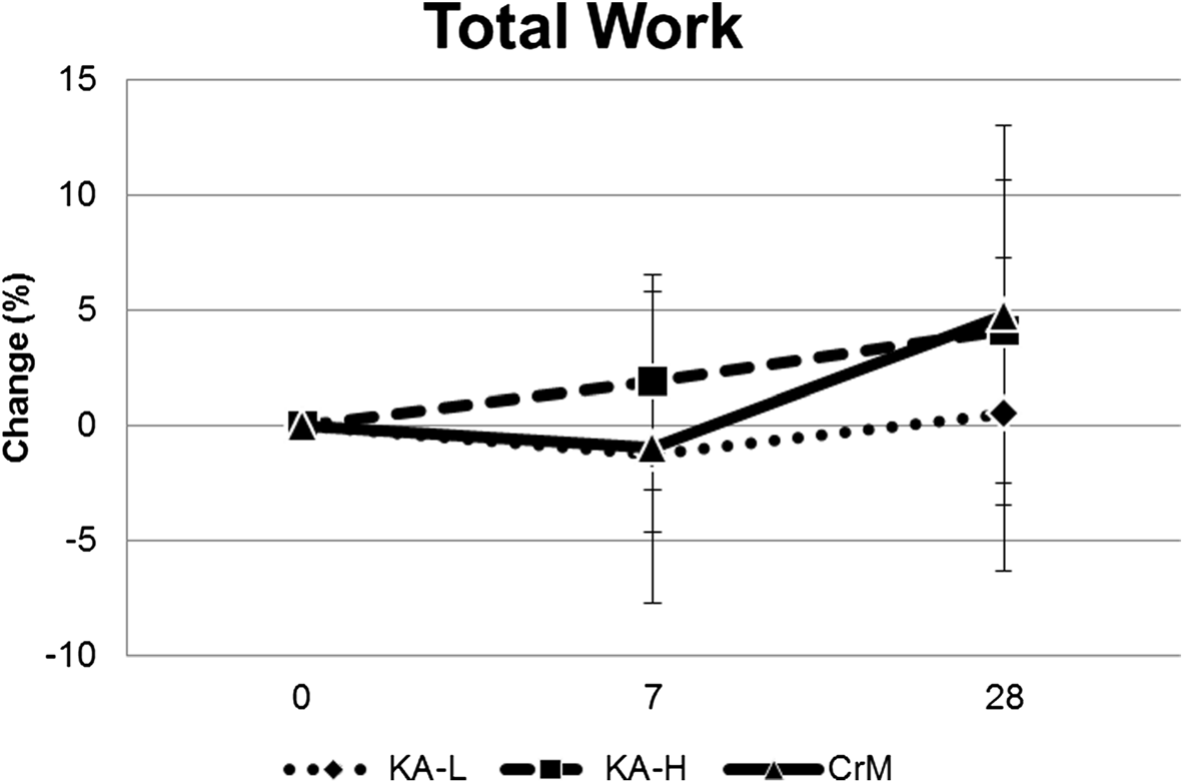
Changes in cycling anaerobic work capacity from baseline.

Table 8 shows upper and lower body 1RM strength data observed for each group while Figure 3 shows the changes in 1RM bench press. There was a significant increase in 1RM for bench press in all groups over time (97.6 ± 22.3 to 101.3 ± 22.6 kg, p < 0.001) with no significant group x time interactions observed among groups in changes in bench press 1RM (KA-L 3.22 ± 1.5, KA-H 3.3 ± 6.8, CrM 4.5 ± 3.7 kg, p = 0.73). There was no significant difference observed in hip sled/leg press 1RM over time (449.5 ± 162, 471.1 ± 167, p = 0.33) or interactions observed among groups in changes in hip sled/leg press 1RM (KA-L 8.7 ± 111, KA-H 68.8 ± 96, CrM −13.3 ± 185 kg, p = 0.33) Table 9 shows results for the anaerobic capacity test while Figure 4 presents changes in total work observed for each group. MANOVA analysis revealed an overall time effect (Wilks’ Lambda p = 0.001) with no significant overall group x time effects (Wilks’ Lambda p = 0.47) in anaerobic capacity variables. Univariate MANOVA analysis revealed that average power (p = 0.005), peak power (p = 0.003), and total work (p = 0.005) increased in all groups over time with no significant group x time interactions observed among groups. Total work performed on the anaerobic capacity sprint test increased in all groups over time (−69 ± 1,030, 552 ± 1,361 J, p = 0.02) with no significant group x time effects observed among groups (KA-L −278 ± 676, 64 ± 1,216; KA-H 412 ± 1,041, 842 ± 1,369; CrM −301 ± 1,224, 775 ± 1,463 J, p = 0.32).

**Table 8:** One Repetition Maximum Strength

**Variable**	**N**	**Group**	**Day**			**p-level**
			**0**	**28**		
**Upper Body** (kg)	12	KA-L	95.3 ± 25.4	98.6 ± 24.7	Group	0.89
	11	KA-H	98.4 ± 18.2	101.7 ± 17.3	Time	0.001
	12	CrM	99.12 ± 24.0	103.7 ± 26.1	G x T	0.73
**Lower Body** (kg)	12	KA-L	445.3 ± 182	454.1 ± 155	Group	0.52
	12	KA-H	465.4 ± 117	539.0 ± 163	Time	0.35
	12	CrM	439.1 ± 189	425.8 ± 175	G x T	0.31

Values are means ± standard deviations. Data were analyzed by MANOVA with repeated measures. Greenhouse-Geisser time and group x time (G x T) interaction p-levels are reported with univariate group p-levels.

**Table 9:** Wingate Anaerobic Sprint Capacity

**Variable**	**N**	**Group**	**Day**				**p-level**
			**0**	**7**	**28**		
**Mean Power** (W)	12	KA-L	658 ± 136	651 ± 134	660 ± 138	Group	0.61
	11	KA-H	689 ± 99	703 ± 113	717 ± 114	Time	0.005
	12	CrM	660 ± 119	652 ± 108	688 ± 105	G x T	0.21
**Peak Power** (W)	12	KA-L	1,274 ± 259	1,393 ± 286	1,585 ± 526	Group	0.50
	11	KA-H	1,329 ± 285	1,538 ± 389	1,616 ± 378	Time	0.003
	12	CrM	1,478 ± 376	1,626 ± 281	1,571 ± 409	G x T	0.48
**Total Work** (J)	12	KA-L	19,728 ± 4,076	19,450 ± 3,910	19,792 ± 4,153	Group	0.59
	11	KA-H	20,681 ± 2,968	21,093 ± 3,387	21,523 ± 3,432	Time	0.005
	12	CrM	19,799 ± 3,564	19,497 ± 3,210	20,573 ± 3,128	G x T	0.22

Values are means ± standard deviations. Data were analyzed by MANOVA with repeated measures. Greenhouse-Geisser time and group x time (G x T) interaction p-levels are reported with univariate group p-levels.

### Clinical chemistry panels

Table 10 presents blood lipid data observed throughout the study Overall MANOVA revealed no time (Wilks’ Lambda p = 0.17) or groups x time effects (Wilks’ Lambda 0.15) in blood lipids. Univariate MANOVA also found no group x time interactions in total cholesterol (TCHL, p = 0.10), high-density lipoprotein (HDL, p = 0.64), the ratio of TCHL to HDL (p = 0.09), and triglycerides (TRIG, p = 0.45). Some group x time effects were observed among groups in low-density lipoprotein (LDL) levels (p = 0.005) with LDL levels significantly decreasing after the loading phase in the CrM group. However, values remained low and near baseline. Univariate ANOVA revealed no significant differences among groups in blood glucose (p = 0.67).

**Table 10:** Serum lipids and glucose

**Marker**	**N**	**Group**	**Day**				**p-level**
			**0**	**7**	**28**		
**TCHL** (mg/dl)	11	KA-L	149.1 ± 25	153.0 ± 23	149.9 ± 28	Group	0.91
	12	KA-H	153.3 ± 26	152.3 ± 28	157.5 ± 22	Time	0.15
	12	CrM	156.3 ± 20	147.3 ± 19	158.9 ± 21	G x T	0.10
**HDL** (mg/dl)	11	KA-L	48.8 ± 11.3	51.0 ± 9.3	52.9 ± 11.4	Group	0.42
	12	KA-H	53.0 ± 16.0	53.9 ± 18.4	53.6 ± 14.4	Time	0.03
	12	CrM	45.6 ± 6.5	47.6 ± 7.3	48.5 ± 8.4	G x T	0.64
**TCHL: HDL Ratio**	11	KA-L	3.16 ± 0.7	3.09 ± 0.6	2.92 ± 0.7	Group	0.34
	12	KA-H	3.03 ± 0.6	2.95 ± 0.5	3.04 ± 0.5	Time	0.04
	12	CrM	3.48 ± 0.6	3.15 ± 0.6	3.36 ± 0.7	G x T	0.09
**LDL**	11	KA-L	83.4 ± 16*	86.5 ± 16	81.4 ± 18*	Group	0.66
(mg/dl)	12	KA-H	79.4 ± 18*	82.7 ± 19	83.7 ± 16*	Time	0.42
	12	CrM	89.8 ± 20	81.4 ± 15†	92.5 ± 17	G x T	0.005
**TRIG** (mg/dl)	11	KA-L	84.5 ± 33	77.3 ± 30	78.5 ± 37	Group	0.20
	12	KA-H	105.1 ± 37	78.4 ± 26	101.1 ± 27	Time	0.07
	12	CrM	104.1 ± 28	92.1 ± 30	89.6 ± 30	G x T	0.45
**Glucose** (mg/dl)	11	KA-L	93.0 ± 5.1	90.5 ± 8.2	93.6 ± 4.7	Group	0.44
	12	KA-H	91.1 ± 6.6	92.7 ± 8.1	90.4 ± 6.9	Time	0.57
	12	CrM	90.5 ± 9.6	89.6 ± 5.5	88.3 ± 6.3	G x T	0.67

Values are means ± standard deviations. Lipid data were analyzed by MANOVA with repeated measures. Greenhouse-Geisser time and group x time (G x T) interaction p-levels are reported with univariate group p-levels. Glucose data were analyzed by repeated measures univariate ANOVA. † represents p < 0.05 difference from baseline. * represents p < 0.05 difference from CrM.

Table 11 shows markers of catabolism and bone status. Overall MANOVA revealed significant time (Wilks’ Lambda p < 0.001) effects with no significant group x time effects (Wilks’ Lambda p = 0.19) in markers of catabolism. Univariate MANOVA found no significant group x time interactions in blood urea nitrogen (BUN, p = 0.75), BUN to creatinine ratio (p = 0.24), aspartate aminotransferase (AST, p = 0.68), alanine aminotransferase (ALT, p = 0.48), total protein (p = 0.84), and total bilirubin (TBIL, p = 0.26). Serum creatinine levels increased in all groups (p < 0.001) over time with a significant group x time interaction demonstrating higher doses of creatine in the CrM and KA-H groups promoting significantly greater increases in serum creatinine (p = 0.03) than the KA-L group. However, creatinine levels in the CrM and KA-H groups were only 0.1 – 0.2 mg/dL greater than the KA-L group, well within normal values for active individuals, and of no clinical significance. MANOVA analysis of bone related markers found no significant time (Wilks’ Lambda p = 0.83) or group x time effects (Wilks’ Lambda p = 0.78). Likewise, univariate MANOVA analysis revealed no significant interactions among groups in bone mineral content (p = 0.66), albumin (ALB, p = 0.89), globulin (GLOB, p = 0.42), the ratio of ALB to GLOB (p = 0.45), calcium (p = 0.76), or alkaline phosphatase (ALK, p = 0.65).

**Table 11:** Markers of catabolism and bone status

**Marker**	**N**	**Group**	**Day**				**p-level**
			**0**	**7**	**28**		
**BUN** (mg/dl)	11	KA-L	16.0 ± 5.3	15.3 ± 4.9	15.6 ± 5.1	Group	0.89
	12	KA-H	16.1 ± 3.3	16.6 ± 3.9	16.6 ± 3.6	Time	0.70
	12	CrM	16.4 ± 3.2	15.7 ± 2.7	16.1 ± 4.7	G x T	0.75
**Creatinine**	11	KA-L	1.04 ± 0.08	1.08 ± 0.11	1.13 ± 0.10†	Group	0.07
(mg/dl)	12	KA-H	1.07 ± 0.14	1.23 ± 0.18†*	1.26 ± 0.13†*	Time	0.001
	12	CrM	1.11 ± 0.19	1.28 ± 0.20†*	1.23 ± 0.15†*	G x T	0.03
**BUN:CRN Ratio**	11	KA-L	15.5 ± 5.1	14.5 ± 5.6	14.1 ± 5.6	Group	0.83
	12	KA-H	15.1 ± 3.4	13.7 ± 3.4	13.3 ± 3.4	Time	0.001
	12	CrM	15.2 ± 3.7	12.4 ± 2.6	13.2 ± 3.8	G x T	0.24
**AST** (U/L)	11	KA-L	25.4 ± 9.6	26.5 ± 8.4	29.5 ± 12.9	Group	0.62
	12	KA-H	27.3 ± 10.5	25.6 ± 8.3	32.0 ± 12.0	Time	0.02
	12	CrM	24.9 ± 7.9	23.8 ± 7.5	26.3 ± 7.8	G x T	0.70
**ALT** (U/L)	11	KA-L	21.5 ± 11.2	23.5 ± 14.2	28.7 ± 19.4	Group	0.50
	12	KA-H	24.1 ± 15.6	22.3 ± 12.2	27.3 ± 9.1	Time	0.05
	12	CrM	21.3 ± 7.34	18.0 ± 4.2	21.3 ± 5.5	G x T	0.48
**Total Protein** (g/dl)	11	KA-L	7.4 ± 0.6	7.4 ± 0.4	7.4 ± 0.4	Group	0.87
	12	KA-H	7.3 ± 0.3	7.3 ± 0.3	7.3 ± 0.2	Time	0.88
	12	CrM	7.3 ± 0.2	7.3 ± 0.2	7.4 ± 0.3	G x T	0.84
**TBIL** (mg/dl)	11	KA-L	0.84 ± 0.7	0.75 ± 0.3	0.76 ± 0.3	Group	0.60
	12	KA-H	0.88 ± 0.5	0.89 ± 0.5	0.77 ± 0.4	Time	0.90
	12	CrM	0.63 ± 0.2	0.71 ± 0.2	0.77 ± 0.2	G x T	0.26
**Bone Mineral**	11	KA-L	2,517 ± 404	2,503 ± 409	2,505 ± 398	Group	0.59
**Content** (g)	12	KA-H	2,632 ± 457	2,604 ± 466	2,615 ± 456	Time	0.49
	12	CrM	2,446 ± 344	2,456 ± 0.2	2,441 ± 351	G x T	0.66
**Albumin** (g/dl)	11	KA-L	4.80 ± 0.3	4.81 ± 0.4	4.81 ± 0.2	Group	0.95
	12	KA-H	4.83 ± 0.2	4.74 ± 0.2	4.78 ± 0.1	Time	0.73
	12	CrM	4.82 ± 0.2	4.80 ± 364	4.79 ± 0.2	G x T	0.89
**Globulin** (g/dl)	11	KA-L	2.60 ± 0.4	2.63 ± 0.3	2.55 ± 0.3	Group	0.90
	12	KA-H	2.56 ± 0.3	2.58 ± 0.2	2.52 ± 0.3	Time	0.85
	12	CrM	2.55 ± 0.3	2.54 ± 0.2	2.62 ± 0.3	G x T	0.42
**Alb:Glob Ratio**	11	KA-L	1.88 ± 0.3	1.85 ± 0.2	1.90 ± 0.2	Group	0.98
	12	KA-H	1.90 ± 0.1	1.86 ± 0.2	1.91 ± 0.1	Time	0.70
	12	CrM	1.88 ± 0.2	1.90 ± 0.2	1.84 ± 0.2	G x T	0.45
**Calcium** (mg/dl)	11	KA-L	9.87 ± 0.5	9.85 ± 0.5	9.76 ± 0.4	Group	0.42
	12	KA-H	9.83 ± 0.2	9.81 ± 0.4	9.84 ± 0.2	Time	0.51
	12	CrM	9.77 ± 0.3	9.63 ± 0.4	9.67 ± 0.3	G x T	0.76
**ALK** (U/L)	11	KA-L	82.0 ± 16.4	84.1 ± 20.5	83.9 ± 17.0	Group	0.88
	12	KA-H	81.1 ± 29.7	83.8 ± 30.3	87.1 ± 27.6	Time	0.29
	12	CrM	78.9 ± 20.7	80.6 ± 26.4	78.8 ± 23.1	G x T	0.65

Values are means ± standard deviations. Data were analyzed by MANOVA with repeated measures. Greenhouse-Geisser time and group x time (G x T) interaction p-levels are reported with univariate group p-levels. † represents p < 0.05 difference from baseline. * represents p < 0.05 difference from KA-L.

Table 12 presents serum electrolyte data. Overall MANOVA analysis revealed a significant time effect (Wilks’ Lambda p = 0.02) with no significant overall interaction (Wilks’ Lambda p = 0.26). Univariate MANOVA analysis revealed some small time effects in chloride levels (p = 0.008) and a trend toward an interaction in potassium levels (p = 0.08) but the small changes observed would have no clinical significance. Finally, Table 12 shows whole blood markers assessed throughout the study. Overall MANOVA revealed no significant time (Wilks’ Lambda p = 0.25) or group x time effects (Wilks’ Lambda p = 0.78). Likewise, no significant interactions were observed among groups in white blood cell count (WBC, p = 0.45), red blood cell count (RBC, p = 0.64), hematocrit (p = 0.65), hemoglobin (p = 0.59), mean corpuscular volume (MCV, p = 0.56), mean corpuscular hemoglobin (MCH, p = 0.44), mean corpuscular hemoglobin concentration (MCHC, p = 0.68), red blood cell distribution width (RBCDW, p = 0.92), or platelet count (p = 0.48).

**Table 12:** Serum electrolyte status

**Marker**	**N**	**Group**	**Day**				**p-level**
			**0**	**7**	**28**		
**Sodium** (mmol/L)	11	KA-L	140.1 ± 2.3	139.9 ± 1.1	140.0 ± 1.3	Group	0.98
	12	KA-H	139.9 ± 2.3	139.7 ± 2.4	140.3 ± 2.1	Time	0.28
	12	CrM	140.8 ± 2.1	139.3 ± 1.4	139.7 ± 1.6	G x T	0.57
**Potassium** (mmol/L)	11	KA-L	4.54 ± 0.3	4.86 ± 0.4	4.82 ± 0.3	Group	0.65
	12	KA-H	4.89 ± 0.5	4.71 ± 0.6	5.00 ± 0.3	Time	0.11
	12	CrM	4.74 ± 0.4	4.93 ± 0.4	4.81 ± 0.4	G x T	0.08
**Chloride** (mmol/L)	11	KA-L	103.3 ± 2.2	103.0 ± 2.4	103.8 ± 1.9	Group	0.21
	12	KA-H	102.4 ± 2.2	101.5 ± 2.2	102.6 ± 2.4	Time	0.008
	12	CrM	104.3 ± 2.2	102.3 ± 1.7	103.1 ± 1.8	G x T	0.21

Values are means ± standard deviations. Data were analyzed by MANOVA with repeated measures. Greenhouse-Geisser time and group x time (G x T) interaction p-levels are reported with univariate group p-levels.

**Table 13:** Whole blood markers

**Marker**	**N**	**Group**	**Day**				**p-level**
			**0**	**7**	**28**		
**WBC** (x10^3^/ul)	9	KA-L	5.73 ± 0.6	6.13 ± 0.5	6.17 ± 1.5	Group	0.95
	12	KA-H	5.83 ± 1.1	5.76 ± 0.9	6.36 ± 1.1	Time	0.16
	12	CrM	5.97 ± 1.2	5.73 ± 1.0	5.98 ± 1.2	G x T	0.45
**RBC** (x10^6^/ul)	9	KA-L	5.44 ± 0.4	5.38 ± 0.5	5.44 ± 0.3	Group	0.28
	12	KA-H	5.10 ± 0.4	5.18 ± 0.3	5.23 ± 0.3	Time	0.91
	12	CrM	5.42 ± 0.5	5.41 ± 0.5	5.35 ± 0.7	G x T	0.64
**Hematocrit** (%)	9	KA-L	48.4 ± 3.4	47.9 ± 4.3	48.1 ± 2.9	Group	0.17
	12	KA-H	46.5 ± 3.2	47.0 ± 2.8	47.4 ± 1.8	Time	0.96
	12	CrM	45.9 ± 2.3	46.1 ± 2.5	45.2 ± 5.4	G x T	0.65
**Hemoglobin** (g/dl)	9	KA-L	16.0 ± 1.6	16.0 ± 1.6	16.0 ± 1.2	Group	0.21
	12	KA-H	15.2 ± 1.2	15.7 ± 1.0	15.6 ± 0.7	Time	0.60
	12	CrM	15.1 ± 0.9	15.2 ± 1.1	14.9 ± 2.0	G x T	0.62
**MCV** (fL)	9	KA-L	89.0 ± 2.8	88.9 ± 2.9	88.3 ± 2.8	Group	0.10
	12	KA-H	91.1 ± 3.5	90.8 ± 3.1	90.7 ± 3.6	Time	0.03
	12	CrM	85.4 ± 9.2	85.7 ± 9.5	85.0 ± 9.1	G x T	0.56
**MCH** (pg/cell)	9	KA-L	29.4 ± 1.5	29.6 ± 1.2	29.3 ± 1.2	Group	0.34
	12	KA-H	29.8 ± 1.6	30.2 ± 1.5	28.4 ± 4.9	Time	0.20
	12	CrM	28.1 ± 3.5	28.3 ± 3.7	27.9 ± 3.3	G x T	0.44
**MCHC** **(g/dl)**	9	KA-L	33.0 ± 1.3	33.3 ± 0.9	33.2 ± 0.9	Group	0.73
	12	KA-H	32.8 ± 0.9	33.3 ± 0.8	32.9 ± 0.6	Time	0.22
	12	CrM	32.9 ± 1.1	32.9 ± 1.3	32.9 ± 0.8	G x T	0.68
**RBCDW** (%)	9	KA-L	13.0 ± 0.5	13.0 ± 0.9	12.9 ± 0.7	Group	0.34
	12	KA-H	13.8 ± 1.1	13.7 ± 1.0	13.5 ± 1.5	Time	0.41
	12	CrM	13.7 ± 1.4	13.7 ± 1.7	13.6 ± 1.6	G x T	0.92
**Platelet Count** (x10^3^/ul)	9	KA-L	266 ± 45	266 ± 52	280 ± 45	Group	0.12
	12	KA-H	253 ± 54	248 ± 62	269 ± 65	Time	0.32
	12	CrM	222 ± 69	222 ± 74	216 ± 65	G x T	0.48

Values are means ± standard deviations. White and red cell whole blood markers were analyzed by MANOVA with repeated measures. Greenhouse-Geisser time and group x time (G x T) interaction p-levels are reported with univariate group p-levels.

## Discussion

The purpose of this study was to determine if supplementing the diet with recommended (1.5 g/d for 28-days) or creatine equivalent loading and maintenance doses of a purported buffered form of creatine (20 g/d for 7-days and 5 g/d for 21-days) was more effective in increasing muscle creatine retention, body composition, strength, and/or anaerobic capacity than supplementing the diet with creatine monohydrate (20 g/d for 7-days and 5 g/d for 21-days). Additionally, the study was undertaken to determine whether supplementing the diet with recommended or equivalent creatine doses of a purported buffered form of creatine was associated with fewer side effects in comparison to creatine monohydrate. Results of the present study clearly show that supplementing the diet with a purported buffered form of creatine is not a more efficacious and/or a safer form of creatine to consume than creatine monohydrate.

According to product claims [28,30], KA is “*up to ten times more powerful than ordinary Creatine*”. The rationale for this contention is based on experiments reported in a patent [29] and/or on the manufacturer’s website [28,30] which indicates that KA has less conversion of creatine to creatinine in fluid over time compared to creatine monohydrate. This is despite the fact that studies show that creatine monohydrate is not significantly degraded to creatinine during the normal digestive process and nearly 99% of creatine monohydrate that is orally ingested is either taken up by tissue or excreted in the urine [1-3,18,21]. Because of this fact, an accepted method of assessing whole body creatine retention has been to subtract daily urinary creatine excretion from daily dietary intake of creatine [32,33,45-47]. Additionally, while it is true that generally the lower the pH and higher the temperature, the greater conversion of creatine to creatinine, studies show that this process takes several days to occur at significant levels even when creatine is exposed to low pH environments [1,19,48]. As described in a recent review [1], the degradation of creatine can be reduced or even halted by either lowering the pH to under 2.5 or increasing the pH. A very high pH results in the deprotonation of the acid group, thereby slowing down the degradation process by making it more difficult for the intramolecular cyclization of creatine to creatinine. However, a very low pH (as is the case in the stomach) results in the protonation of the amide function of the creatine molecule, thereby preventing the intramolecular cyclization of creatine to creatinine [1]. This is the reason that the conversion of creatine to creatinine in the gastrointestinal tract has been reported to be minimal regardless of transit time [7,18,20]. Thus, on the surface, the KA manufacturer’s claims that creatine monohydrate is degraded to creatinine in large amounts after oral ingestion and that a “*buffered*” or “*pH-correct*” would significantly reduce this effect once consumed and thereby promote greater uptake of creatine in the muscle is inconsistent with available literature on creatine [1].

Results of the present study do not support claims that a large amount of creatine monohydrate was converted to creatinine during the digestive process and thereby resulted in less of an increase in muscle creatine than KA. In this regard, while serum creatinine levels increased to a greater degree in the KA-H and CrM groups that ingested larger amounts of creatine, the 0.1 - 0.2 mg/dL greater increase observed in creatinine compared to the KA-L group was well within normal limits (i.e., <1.28 ± 0.20 mg/dl) particularly for resistance-trained males. Therefore, this small change would be clinically insignificant. Additionally, a significant increase from baseline in serum creatinine was also observed in the KA-L and KA-H groups despite claims that KA completely prevents the conversion of creatine to creatinine. These findings do not support contentions that CrM is degraded to creatinine in large amounts or that KA is not converted to creatinine at all.

Previous research has shown that ingestion of 20 g/d of CrM for 5–7 days can increase muscle creatine content 10-40% after 5–7 d of supplementation [1,4-8,10]. Prolonged low-dose ingestion of CrM (e.g., 2 – 3 g/d for 4–6 weeks) has also been reported to increase muscle creatine content in a similar manner as loading strategies [4,7,8]. The manufacturer of KA claims that ingesting 1.5 g of KA is equivalent to ingesting 10–15 g of CrM [28]. If this were true, those ingesting recommended levels of KA (1.5 g/d for 28-days) should experience a similar increase in muscle creatine as those participants ingesting recommended loading (20 g/d for 7-days) and maintenance doses (5 g/d for 21-days) of CrM. Results of the present study indicated that supplementing the diet with manufacturer’s recommended levels of KA (1.5 g/d) did not increase muscle free creatine content to the same degree as loading and maintenance doses of CrM. In fact, although no overall group effect was observed among the three groups studied (p = 0.14), pairwise comparison of the mean group change from baseline in the KA-L group was 11 times less than the change observed following CrM supplementation (KA-L −1.1 ± 4.3, CrM 11.2 ± 4.3 mmol/kg DW [mean ± SEM], p = 0.053). After 28-days of supplementation, muscle free creatine content in the KA-L group was increased by 4.71 ± 27.0 mmol/kg DW compared to 22.3 ± 21.0 mmol/kg DW in the CrM group representing a 4.7 fold less effect of KA supplementation than CrM when comparing recommended levels. Consequently, results of the present study do not support claims that ingesting 1.5 grams of KA is as effective as ingesting 10–15 grams of creatine monohydrate. Even when participants ingested creatine equivalent amounts of KA and CrM (i.e., 20 g/d for 7-days and 5 g/d for 21-days), KA did not promote greater increases in muscle free creatine. In fact, while not significantly different, changes in muscle creatine in the KA-H group were more than two times less than the changes observed in the CrM group (KA-H 9.07 ± 23.2; CrM 22.3 ± 21.0 mmol/kg DW). Thus, results of the present study do not support claims that ingesting a purported buffered form of creatine is more effective in increasing muscle creatine content than creatine monohydrate.

While some may argue that since there is generally large variability in measuring muscle phosphagen levels and we were unable to obtain reliable PCr measurements, it is difficult to make a definitive conclusion about the effects of KA on muscle creatine content based on measuring muscle free content alone. However, present findings also provide no support for claims that KA supplementation is “*up to ten times more powerful than ordinary Creatine*.” In this regard, while time effects were observed in training adaptations, supplementing the diet with KA (at recommended or creatine equivalent loading and maintenance doses) did not promote statistically greater gains in fat free mass, 1 RM strength, or anaerobic sprint performance capacity compared to CrM. At best, one can conclude that ingesting recommended and creatine equivalent loading and maintenance amounts of KA resulted in similar training adaptations as creatine monohydrate supplementation at recommended loading and maintenance levels. However, results of the present investigation provide no evidence to support claims that KA is “*the world’s most potent creatine*” [28].

Further, results of the present investigation provided no evidence that KA is a safer form of creatine to consume at either lower recommended levels or higher creatine equivalent doses compared to normal loading and maintenance doses of creatine monohydrate. In this regard, there were no significant differences observed among groups in BIA determined total body water or serum electrolyte status. Likewise, no cramping or other side effects were reported. These findings are consistent with previous studies that have indicated that creatine supplementation does not promote dehydration and/or cramping [9,21-26]. There were also no significant differences observed among groups in serum lipids (TCHL, HDL, TCHL:HDL ratio, TRIG) or blood glucose. Serum LDL decreased slightly in response to creatine loading in the CrM group but returned to baseline after ingesting maintenance doses of CrM suggesting these changes were transient. Additionally, no significant differences were observed among groups in markers of catabolism (BUN, BUN:CRN, AST, ALT, Total Protein, TBIL), markers of bone status (bone mineral content, ALB, GLOB, ALB:GLOB, calcium, ALK) or whole blood markers (WBC, RBC, Hematocrit, Hemoglobin, MCV, MCH, MCHC, RBCDW, platelet counts). Moreover, values remained within normal levels for active individuals. These findings are consistent with other studies that have examined the safety of creatine supplementation in active individuals [1,3,21,26,27,38]. Consequently, present findings do not support claims that KA is a safer form of creatine to ingest than creatine monohydrate.

## Conclusion

In summary, supplementation of the diet with recommended doses of a purported buffered form of creatine (1.5 g/d) for 28-days or equivalent loading (20 g/d for 7-days) and maintenance doses (5 g/d for 21-days) of CrM did not promote greater increases in muscle creatine content or training adaptations in comparison to creatine monohydrate (20 g/d for 7-days, 5 g/d for 21-days). Additionally, there was no evidence to support claims that the buffered form of creatine was associated with fewer side effects or was a safer form of creatine to consume than creatine monohydrate. While it could be argued that supplementing the diet with any form of creatine may provide some health and/or ergogenic benefits over time as long as it delivers sufficient amounts of creatine to increase muscle creatine content; present findings do not support claims that KA is a more efficacious and/or safer form of creatine than creatine monohydrate. With this said, some limitations of this study should be noted. For example, this study did not have a control group and depended on participants to self-report side effects. Therefore, while the safety profile of short and long-term creatine monohydrate supplementation has been well established, safety and efficacy could only be compared to ingesting different levels and forms of creatine and not controls. There is also variability in conducting muscle and blood assays as well as variability in conducting performance tests. In some instances, large mean differences among groups were either not statistically significant or only approached significance. It is possible that some of these differences would have been significant if a control group was included in the study design and/or more subjects were studied to increase statistical power. Nevertheless, results from the present study do not support claims that KA is a more efficacious and/or safer form of creatine to consume than creatine monohydrate.

## Competing interests

AlzChem AG (*Trostberg, Germany*) provided funding for this study through a research grant to Texas A&M University. All researchers involved independently collected, analyzed, and interpreted the results from this study and have no financial interests concerning the outcome of this investigation. RBK has received grants as Principal Investigator through institutions with which he has been affiliated to conduct exercise and nutrition related research, has served as a legal and scientific consultant, and currently serves as a scientific consultant for Woodbolt International (*Bryan, TX*). Remaining coauthors have no competing interests to declare. Data from this study have been presented at the International Society of Sports Nutrition Annual meeting and have not been submitted for publication to any other journals. Publication of these findings should not be viewed as endorsement by the investigators or their institutions of the nutrients investigated.

## Authors’ contributions

ARJ served as the study coordinator, oversaw all testing, and assisted in data analysis and writing of the manuscript. JMO assisted in data collection and statistical analysis. AS assisted with data collection. EG assisted with data collection and reviewed and approved nutritional records as the studies’ registered dietitian. JF and SR supervised the biopsy procedures. MG assisted in experimental design, data analysis, and manuscript preparation. KK supervised muscle assays and CM served as a collaborating scientist. CR served as lab coordinator and oversaw data collection and quality control of the study. RBK served as Principal Investigator and contributed to the design of the study, statistical analysis, manuscript preparation, and procurement of external funding. All authors read and approved the final manuscript.

## Funding

Supported by AlzChem AG, Germany.
